# Influence of SARS-CoV-2 reinfection on bone health in the population over 55 years old in Beijing, China: a cross-sectional study

**DOI:** 10.3389/fpubh.2026.1758835

**Published:** 2026-02-19

**Authors:** Qian Qiu, Tong Wu, Yuyong Jiang, Zimeng Shang, Wanxin Shi, Xiaohui Zhang, Xu Han, Zhiyun Yang, Xieyuan Jiang, Xiuying Liu

**Affiliations:** 1 Beijing Center for Diseases Prevention and Control, Beijing, China; 2 Capital Medical University, Beijing, China; 3 Center of Integrative Medicine, Beijing Ditan Hospital, Capital Medical University, Beijing, China; 4 Cuigezhuang Community Health Service Center, Beijing, China; 5 Department of Orthopedic Trauma, Beijing Jishuitan Hospital, Capital Medical University, Beijing, China; 6 Beijing Research Institute of Traumatology and Orthopaedics, Beijing, China

**Keywords:** aging, bone mineral density, influencing factors, osteoporosis, SARS-CoV-2

## Abstract

**Background:**

Despite the global prevalence of SARS-CoV-2 reinfection, relatively little is known regarding its association with osteoporosis in older adults. This study investigated the potential impact of severe acute respiratory syndrome coronavirus 2 (SARS-CoV-2) infection on bone health or osteoporosis risk in adults aged 55 years and older in Beijing, China, while also exploring additional factors that may influence this association.

**Methods:**

This cross-sectional study was conducted at Beijing Ditan Hospital, Beijing, China, between August and December 2024. Patients aged ≥ 55 years with SARS-CoV-2 infections were included. Data on demographic characteristics, lifestyle habits, diet, serum contents of 25-hydroxyvitamin D (25-OH-VD), and other potential risk factors for osteoporosis were collected through structured questionnaires. Bone mineral density (BMD) was assessed using dual-energy X-ray absorptiometry (DXA). To identify the variables associated with osteoporosis, logistic regression analysis was employed.

**Results:**

Three hundred and eighty-eight patients were enrolled, comprising 272 individuals with a single SARS-CoV-2 infection and 116 with two or more infections. The results revealed that individuals who had reinfection revealed a considerably higher prevalence of osteoporosis (48.3% vs. 17.3%, *p* < 0.0001). This trend was consistent across different skeletal sites: 33.6% vs. 7.4% at the left hip, 37.9% vs. 5.5% at the right hip (*p* < 0.0001), and 30.2% vs. 12.9% at the lumbar spine (L1–L4) (*p* < 0.0001). SARS-CoV-2 reinfection remained substantially associated with osteoporosis after controlling for con-founding variables, with an OR of 3.08 (95% CI: 1.696–5.593).

**Conclusion:**

In summary, the risk of osteoporosis among individuals with SARS-CoV-2 reinfection should be carefully monitored, and greater emphasis should be placed on early, individualized prevention and treatment strategies.

## 1 Introduction

Osteoporosis, characterized by decreases in bone mineral density (BMD) and reduced bone strength, is a prevalent health concern worldwide as the aging population grows (1). In the USA, over 40 million older adults have osteopenia, and in Australia, 66% of adults over the age of 50 have osteoporosis (2, 3). According to data from China’s first osteoporosis epidemiological survey, 32.0% of people over 65 had osteoporosis, while 46.4% of people over 50 had low bone mineral density (4). Osteoporosis is becoming more common in China as the country’s population ages. By 2050, 212 million people in China are expected to have osteoporosis (5). Osteosarcopenia is linked to raised risks of falls, fractures, functional disability, and mortality, according to a population-based prospective cohort investigation conducted in Chile (6). Osteoporosis prevention in older adults is a major public health priority due to the substantial increase in disease burden and healthcare expenditures associated with osteoporosis (7).

Severe acute respiratory syndrome coronavirus 2 (SARS-CoV-2) is the pathogen responsible for coronavirus disease 2019 (COVID-19), which has over 770 million confirmed cases as of 2025 (8). Bone metabolism is significantly impacted by SARS-CoV-2 infections, as indicated by several studies. Two weeks following SARS-CoV-2 infection, significantly enhanced osteoclastogenesis and bone loss was observed in an animal model (9), while recovering patients showed a variety of skeletal abnormalities, such as osteoporosis and decreased BMD, according to Tangl (10). According to numerous studies, SARS-CoV-2 causes inflammatory cytokine dysregulation, which in turn stimulates osteoclast activity and leads to bone loss and resorption in patients with severe infections, particularly inolder individuals (11–13). With the appearance of immune-evading variants and waning immunity, SARS-CoV-2 reinfections have become increasingly common, affecting millions worldwide, particularly among the older population (14). Previous studies have demonstrated that reinfection significantly amplifies higher rates of severe outcomes (hospitalization, mortality) compared to primary infection (15), with emerging evidence suggesting cumulative damage to musculoskeletal health, indicating that repeated inflammatory insults and cytokine dysregulation from multiple infections could cumulatively impair bone remodeling. It is essential to emphasize the connection between SARS-CoV-2 reinfection and osteoporosis in older populations, as most SARS-CoV-2-infected individuals are of older age (14), who are at a higher risk of reinfection.

Osteoporosis risk is influenced by several factors. Individual characteristics such as advancing age, female sex, particularly postmenopausal status, and Caucasian ethnicity have long been linked to increased likelihood of developing osteoporosis (16). This risk can be amplified by nutritional inadequacies, particularly insufficient consumption of vitamin D and calcium. Regular physical exercise has been shown to strengthen bones and help prevent age-related declines in BMD (16, 17).

Identifying these risk factors is crucial for identifying individuals at high risk, enhancing diagnostic accuracy, informing further evaluations, and implementing timely and effective interventions. However, despite the global prevalence of SARS-CoV-2 reinfection, relatively little is known regarding its association with osteoporosis in older adults. It is thus important to understand the influence of reinfection on bone health among older people. Such insights could contribute to personalized prevention and therapeutic strategies for reinfection-associated osteoporosis, ultimately helping to reduce the growing disease burden worldwide.

The influence of SARS-CoV-2 reinfection and other contributing factors on osteoporosis in adults aged ≥ 55 was investigated through a cross-sectional design. The objective was to elucidate the relationship between SARS-CoV-2 reinfection and osteoporosis in the older population, aim to help identify high-risk individuals, promote early and personalized prevention and treatment strategies, and ultimately contribute to reducing the disease burden of osteoporosis in older individuals with COVID-19.

## 2 Methods

### 2.1 Study participants

This cross-sectional investigation was undertaken in Beijing, China, between August and December 2024. The Beijing Ditan Hospital’s Ethics Review Committee at Capital Medical University provided ethical approval (No. [2024]-(083)-01). Written informed consent was obtained from all participants, and their information was kept completely private. The Strengthening the Reporting of Observational Studies in Epidemiology (STROBE) principles were observed. Consecutive eligible patients who attended outpatient clinics of Beijing Ditan Hospital between August and December 2024 were invited to participate.

*Inclusion criteria:*

1. aged 55 years and older;
2. confirmed antigen positive of SARS-CoV-2 for at least one time within the surrounding communities of Beijing Ditan Hospital, Capital Medical University, between December 1, 2019, and August 31, 2024; and
3. willingness to participate with a signed informed permission form.

*Exclusion criteria:*

1. coexisting endocrine disorders such as parathyroid, gonadal, adrenal, or thyroid diseases;
2. immune-related diseases, including rheumatoid arthritis;
3. digestive or renal disorders that could impair calcium or vitamin D absorption and metabolism;
4. neuromuscular diseases, multiple myeloma, or other malignancies;
5. congenital or acquired abnormalities affecting bone metabolism;
6. long-term use of glucocorticoids; and
7. ongoing chemotherapy or use of medications known to influence bone metabolism.

### 2.2 Data collection

Outpatient physicians or nurses who have received training for this study conducted face-to-face surveys to collect information on participants’ demographic features (e.g., age, sex), lifestyle practices (e.g., physical activity), dietary factors (e.g., calcium supplementation), laboratory results for serum 25-hydroxyvitamin D (25-OH-VD), history of SARS-CoV-2 infection, and other potential osteoporosis-related risk factors.

Moreover, the BMD was assessed for each participant by professional radiologists in the hospital, using a Hologic Discovery dual-energy X-ray absorptiometry (DXA) system at the lumbar spine (L1–L4), left hip, and right hip, and daily calibration was conducted according to manufacturer guidelines. T scores of BMD were calculated using data on healthy Chinese as a reference according to the standards of the WHO, and osteoporosis was diagnosed according to the T-score (18):

① Normal: > − 1;
② Osteopenia: between −2.5 and −1;
③ Osteoporosis: < −2.5.

A participant was classified as having osteoporosis if T-scores at any of the measured sites (lumbar spine, left hip, or right hip) were below −2.5. Individuals with two or more documented SARS-CoV-2 infections occurring ≥90 days after the first confirmed infection were classified as having reinfection.

### 2.3 Statistical analysis

Data were analyzed using SPSS version 18.0 (SPSS Inc., Chicago, IL, United States). Categorical variables are represented with 95% confidence intervals (95% CIs) and were compared using Pearson’s χ^2^ test, χ^2^ test for trend, or Fisher’s exact test, as appropriate. Continuous variables are shown as mean ± standard deviation (x ± s) and were compared using t-tests. Logistic regression was utilized to determine variables independently linked to osteoporosis. Statistical significance was defined as a two-tailed *p*-value of less than 0.05.

## 3 Results

### 3.1 Baseline information on participants

Three hundred and eighty-eight participants were including, with all undergoing DXA assessments; the mean age was 64.0 ± 5.7 years and 243 (62.6%) were male. Of the total participants, 272 had a single SARS-CoV-2 infection, while 116 experienced two or more infections. Participants with reinfection were significantly older than those with a single infection (65.5 ± 6.4 *vs.* 63.4 ± 5.3 years, *p* = 0.0023), with a markedly higher number of female in the reinfected group (70.7% *vs.* 59.2%, *p* = 0.0321). In contrast, participants with a single infection reported a higher rate of calcium supplementation (26.8% *vs.* 13.8%, *p* = 0.0051). They were more likely to engage in at least 30 min of daily physical activity (76.8% *vs.* 56.9%, *p* < 0.0001), as illustrated in Table 1.

**Table 1:** Baseline information on study participants.

Baseline information	Total (*N* = 388)	Infection (*N* = 272)	Reinfection (*N* = 116)	*p*
Age (years), mean ±SD	64.0 ± 5.7	63.4 ± 5.3	65.5 ± 6.4	0.0023
Age group (years), N (%)				
55–59	84(21.6)	63(23.2)	21(18.1)	0.0009
60–64	137(35.3)	108(39.7)	29(25.0)	
65–69	99(25.5)	65(23.9)	34(29.3)	
≥70	68(17.5)	36(13.2)	32(27.6)	
Sex, N (%)				
Male	145(37.4)	111(40.8)	34(29.3)	0.0321
Female	243(62.6)	161(59.2)	82(70.7)	
Drink, N (%)	125(32.2)	88(32.4)	37(31.9)	0.9298
Smoke, N (%)	91(23.5)	75(27.6)	16(13.8)	0.0034
25-OH-VD < 20 ng/ml, N (%)	206(53.1)	136(50.0)	70(60.3)	0.0616
Calcium intake, N (%)	89(22.9)	73(26.8)	16(13.79)	0.0051
Daily exercise ≥30 min, N (%)	275(70.9)	209(76.8)	66(56.9)	<0.0001

### 3.2 BMD between the single-infected and reinfected participants

The mean (SD) BMD T score at the left hip was −1.0 (0.8) for individuals with a single SARS-CoV-2 infection and −1.6 (1.1) for those with reinfection (*p* < 0.0001). Similarly, mean T score values were −1.0 (0.9) and −1.5 (1.2) at the right hip (*p* < 0.0001), and −1.1 (1.2) and −1.7 (1.3) at the lumbar spine (L1–L4), respectively (Table 2).

**Table 2:** BMDs and osteoporosis in different parts of the single-infected and reinfected participants.

BMD	Infection	Reinfection	*p*
BMD T score, mean (95%CI)			
Left hip	−1.0 (−1.1 ~ −0.9)	−1.6 (−1.8 ~ −1.4)	<0.0001
Right hip	−1.0 (−1.1 ~ −0.9)	−1.5 (−1.7 ~ −1.3)	<0.0001
Lumbar spine (L1–L4)	−1.1 (−1.2 ~ −0.9)	−1.7 (−1.9 ~ −1.4)	<0.0001
Osteoporosis, % (95%CI)			
Total	17.3 (12.8–21.8)	48.3 (38.9–57.7)	<0.0001
Left hip	7.4 (4.6–11.1)	33.6 (25.1–43.0)	<0.0001
Right hip	5.5 (3.1–8.9)	37.9 (29.1–47.4)	<0.0001
Lumbar spine (L1–L4)	12.9 (9.1–17.4)	30.2 (22.0–39.4)	<0.0001

The overall osteoporosis prevalence was found to be significantly higher among reinfected individuals (48.3%; 95% CI: 38.9–57.7%) compared with those infected only once (17.3%; 95% CI: 12.8–21.8%) (*p* < 0.0001). The pattern remained consistent for all sites: left hip, 33.6% (95% CI: 25.1–43.0%) *vs.* 7.4% (95% CI: 4.6–11.1%) (*p* < 0.0001); right hip, 37.9% (95% CI: 29.1–47.4%) *vs.* 5.5% (95% CI: 3.1–8.9%) (*p* < 0.0001); and lumbar spine (L1–L4), 30.2% (95% CI: 22.0–39.4%) *vs.* 12.9% (95% CI: 9.1–17.4%) (*p* < 0.0001) (Table 2).

However, the two groups were comparable in terms of cervical degenerative changes, alterations in cervical curvature, cervical osteophytes, thoracic wedge deformities, thoracic degenerative changes, thoracic osteophytes, lumbar degenerative changes, lumbar spinal stenosis, or lumbar scoliosis.

### 3.3 Osteoporosis by demographic factors

#### 3.3.1 Osteoporosis in different age subgroups

**Figure 1:**
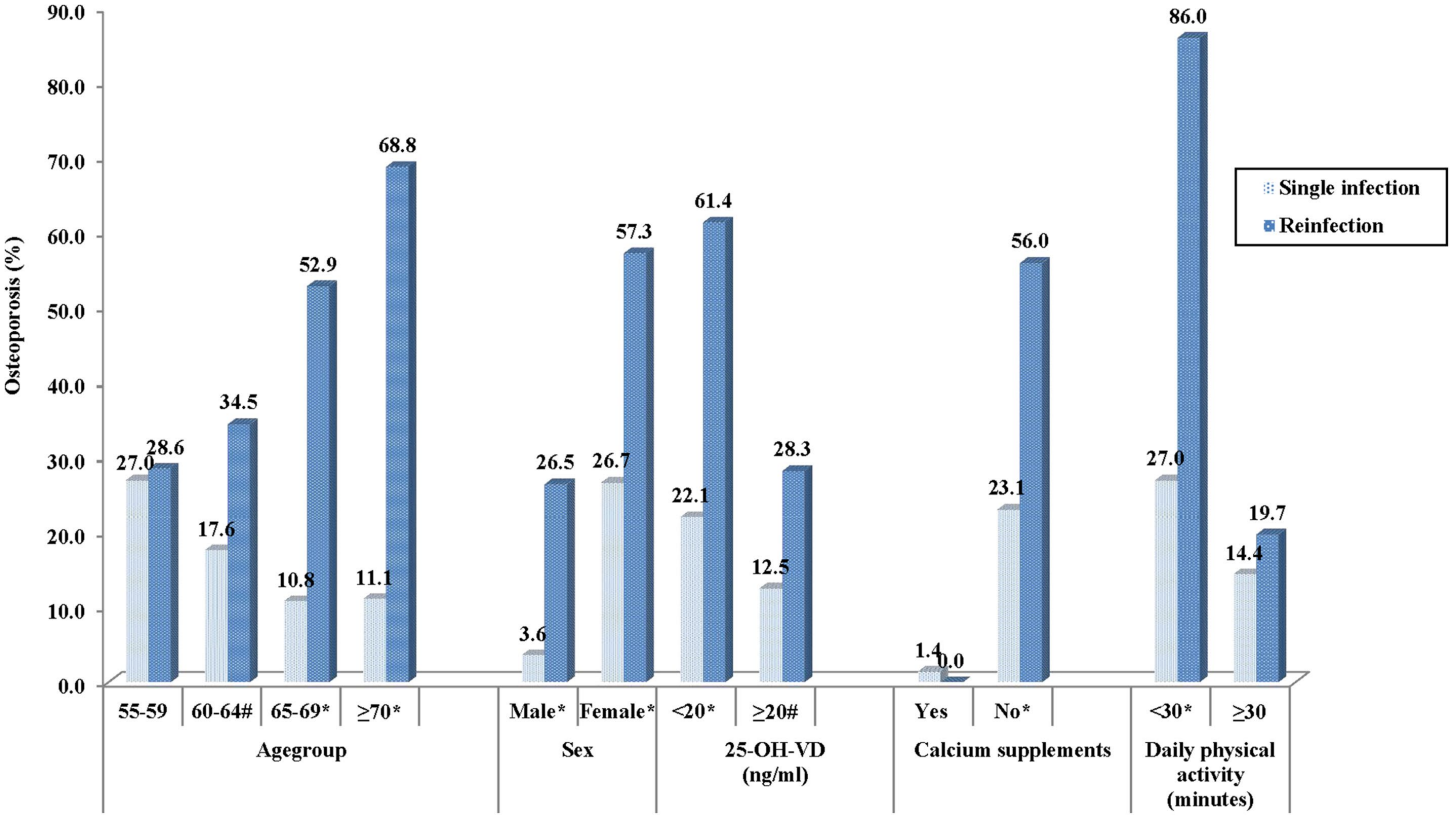
Prevalence of osteoporosis by different demographic factors. ^#^*p* < 0.05 between single infection and reinfection. *p < 0.001 between single infection and reinfection.

There was a marked increase in osteoporosis prevalence with age in reinfected patients (*p* = 0.011), while no significant age-related trend was observed in the single-infection group (*p* = 0.0705). When compared to those with a single infection, the proportion of osteoporosis in the reinfected group was significantly greater among those aged 60–64, 65–69, and ≥70 years (*p* = 0.048, *p* < 0.0001, and *p* < 0.0001, respectively). In comparison, the two groups were essentially comparable in the age range of 55–59 (Figure 1).

Significant sex-related differences in osteoporosis prevalence were observed between the two groups. In both the single-infection and reinfection groups, females demonstrated a substantially higher prevalence of osteoporosis compared with males (*p* < 0.0001 and *p* = 0.0025, respectively). In addition, compared to individuals who were infected only once, those who had reinfection had a noticeably higher percentage of osteoporosis in both sexes (*p* < 0.0001) (Figure 1).

#### 3.3.2 25-OH-VD levels in osteoporosis

In both the single-infection (*p* = 0.0371) and reinfection (*p* = 0.0005) groups, participants with 25-OH-VD deficiency (<20 ng/mL) displayed a substantially greater prevalence of osteoporosis compared to those with sufficient 25-OH-VD levels (≥20 ng/mL). Notable differences in osteoporosis prevalence were also observed between single-infected and reinfected individuals. Among participants with 25-OH-VD deficiency, those with a single infection had a markedly lower prevalence of osteoporosis compared with reinfected individuals (*p* < 0.0001). Similarly, in participants with sufficient 25-OH-VD levels, the single-infection group also demonstrated a significantly lower prevalence of osteoporosis (*p* = 0.0128) (Figure 1).

#### 3.3.3 Calcium supplements for osteoporosis

In this study, 89 participants reported daily calcium supplementation, and only one of them, with a single SARS-CoV-2 infection, was diagnosed with osteoporosis. In contrast, among participants who did not take calcium supplements, osteoporosis prevalence was markedly greater, at 23.1% in the single-infection group and 56.0% in the reinfection group (Figure 1).

#### 3.3.4 Physical activity on osteoporosis

Participants who engaged in a minimum of 30 min of physical activity daily had a significantly lower prevalence of osteoporosis compared with those who exercised for less than 30 min in both groups (single infection: *p* = 0.0201; reinfection: *p* < 0.0001).

Among individuals with less than 30 min of daily activity, the reinfected group exhibited a markedly higher proportion of osteoporosis (*p* < 0.0001). In contrast, among participants who exercised for 30 min or more per day, the prevalence of osteoporosis was comparable between the two groups (*p* = 0.2975). Detailed findings are shown in Figure 1.

### 3.4 Factors influencing osteoporosis

Logistic regression was utilized for the identification of factors linked to osteoporosis. Females had an elevated risk of osteoporosis (OR = 6.632, 95% CI: 3.194–13.770). In contrast, engaging in at least 30 min of daily physical activity (OR = 0.184, 95% CI: 0.098–0.346) and calcium supplementation (OR = 0.025, 95% CI: 0.003–0.193) both demonstrated an association with a diminished risk. After adjusting for potential confounding variables, SARS-CoV-2 reinfection remained a strong independent risk factor for osteoporosis (OR = 3.080, 95% CI: 1.696–5.593) (Table 3).

**Table 3:** Logistic regression determination of factors influencing osteoporosis.

Predictor	Estimate	SE	Waldχ^2^	*p*	Odds ratio	95% CI	
						Lower	Upper
Age group (years)							
55–59	–	–	–	–	Ref	–	–
60–64	−0.2283	0.233	0.9600	0.3272	0.834	0.385	1.805
65–69	−0.0924	0.2591	0.1271	0.7215	0.955	0.414	2.206
≥70	0.3673	0.2870	1.6372	0.2007	1.513	0.610	3.753
Sex							
Male	–	–	–	–	Ref	–	–
Female	1.8919	0.3728	25.7598	<0.0001	6.632	3.194	13.77
Infected times							
1	–	–	–	–	Ref	–	–
≥2	1.1248	0.3044	13.6515	0.0002	3.080	1.696	5.593
Serum 25–OH–VD (ng/ml)							
≥20	–	–	–	–	Ref	–	–
<20	−0.0128	0.3246	0.0016	0.9685	0.987	0.523	1.865
Daily physical activity (minutes)							
<30	–	–	–	–	Ref	–	–
≥30	−1.6936	0.3219	27.6723	<0.0001	0.184	0.098	0.346
Calcium supplement							
No	–	–	–	–	Ref	–	–
Yes	−3.6746	1.0366	12.5669	0.0004	0.025	0.003	0.193

## 4 Discussion

Despite the global prevalence of SARS-CoV-2 reinfection, relatively little is known regarding its association with osteoporosis in older adults. This investigation was undertaken to further investigated the prevalence of osteoporosis between older individuals with single SARS-CoV-2 infection and those with reinfection, providing a basis for subsequent research on the impact of SARS-CoV-2 on bone health, offering valuable insights into the prevention of osteoporosis among individuals aged 55 years and above. Our findings revealed that 48.3% of older participants with SARS-CoV-2 reinfection were diagnosed with osteoporosis, a significantly higher proportion compared with those who had a single infection, even after adjusting for the other influence factors. Moreover, the prevalence of osteoporosis was consistently higher across all measured skeletal sites in the reinfected group. These results indicate that older adults (≥55 years) who have experienced SARS-CoV-2 reinfection are at a heightened risk of osteoporosis. Given that the older population is particularly susceptible to osteoporosis, we recommend effective prevention and management strategies for older adults who have been infected with SARS-CoV-2, especially for those with reinfection, should be implemented to mitigate its adverse effects on bone health and reduce the broader public health burden.

Earlier studies indicate that infection with SARS-CoV-2 adversely affects bone metabolism, contributing to the development of osteoporosis (19). In 2021, Awosanya et al. demonstrated in a SARS-CoV-2 mouse model that infected mice exhibited a marked increase in osteoclast formation compared with uninfected controls, resulting in significant bone loss following infection (9). Similarly, it was found that recovering patients exhibited skeletal abnormalities, such as decreased bone density and osteonecrosis (10). A recent study revealed that the ongoing impact of the COVID-19 pandemic had led to an overall downward trend in BMD (20). Consistent with these findings, our study revealed that osteoporosis was more prevalent in individuals infected with SARS-CoV-2 compared with data from the China Osteoporosis Prevalence Study in older populations (21). This bone loss may be explained by cytokine dysregulation induced by SARS-CoV-2, as proinflammatory factors not only induce differentiation of osteoclasts within bone but also promote inflammation in the skeletal system, thereby enhancing osteoclastogenic activity (11–13).

The so-called “cytokine storm” in COVID-19 is considered a major contributor to disease severity, characterized by increased production of cytokines, such as IL-1, IL-6, IL-17, and TNF-*α* (11, 22). Among these, IL-1 is a well-characterized inflammatory mediator that stimulates osteoclast formation and suppresses bone synthesis through direct activation of the RANK-mediated signaling pathway, resulting in extensive bone and cartilage degradation (23, 24). TNF-α serves as a positive regulator of osteoclastogenesis by inhibiting osteoblastic activity and promoting osteoclastic proliferation and differentiation (25). IL-6 also contributes to bone resorption by inducing osteoclast formation and enhancing their activity (25, 26). Moreover, Th17 cells modultate osteoclastogenesis via IL-17-induced upregulation of RANKL and may participate in fracture healing via their modulatory effects on bone cells (27).

A comprehensive cohort investigation of the consequences of SARS-CoV-2 reinfection found that, relative to individuals who were either uninfected or infected only once, those with reinfection showed a significantly higher risk of severe illness (15). Similarly, Elmedany et al. reported that the severity of SARS-CoV-2 infection was inversely correlated with BMD; patients with severe infections experienced a greater reduction in BMD compared with those with mild or moderate disease (28). These findings suggest that repeated SARS-CoV-2 infections may heighten the risk of osteoporosis as disease severity increases, aligning with the results of our study. One potential mechanism involves the marked elevation of osteoprotegerin (OPG), a key regulator of bone remodeling, observed in patients with severe infections (29).

Amid the global prevalence of SARS-CoV-2 reinfections, close monitoring of bone health in older individuals reinfected with SARS-CoV-2 is critical to prevent or mitigate bone loss and reduce the risk of adverse outcomes, such as fractures associated with osteoporosis.

It is well established that advanced age, female sex, inadequate consumption of calcium and vitamin D, and limited physical activity are major risk factors for osteoporosis (16, 30, 31). It is well-documented that the prevalence of osteoporosis and other bone metabolism disorders increases with age, and that females, regardless of age group, are disproportionately affected (32). The development of osteopenia or osteoporosis in postmenopausal women is also a consequence of estrogen deficiency (33). Consistent with previous research, our study also demonstrated that female participants with SARS-CoV-2 infection had more than a fivefold higher risk of developing osteoporosis compared with males. These results demonstrate the vital role of closely monitoring bone health in older adults, particularly among women.

Moreover, there are several reports of a modest but meaningful link between low calcium intake and reduced BMD (34). Vitamin D deficiency is also associated with a greater risk of osteoporosis, primarily through mechanisms such as increased bone turnover, impaired calcium absorption, secondary hyperparathyroidism, and compromised immune regulation, all of which contribute to bone mass loss (35, 36). Furthermore, insufficient physical activity has been positively correlated with an elevated likelihood of osteoporosis in multiple studies (37).

In agreement with these findings, a higher prevalence of osteoporosis was observed among participants with vitamin D deficiency, inadequate calcium intake, or less than 30 min of daily physical activity. Importantly, even among those with SARS-CoV-2 reinfection, regular calcium supplementation and engaging in at least 30 min of daily physical activity remained protective against osteoporosis. Therefore, maintaining optimal vitamin D levels, ensuring adequate calcium intake, and adopting a physically active lifestyle, along with sufficient exposure to sunlight, are all essential strategies for preventing osteoporosis.

This study has several limitations. First, all individuals were enrolled from the single-center hospital, selection bias may over represent individuals with health issues. Second, information regarding SARS-CoV-2 infection history, lifestyle habits, and dietary intake was collected through face-to-face questionnaires, which made the data susceptible to recall bias. Thirdly, osteoporosis is a multifactorial condition influenced by numerous variables; however, not all potential confounders identified in previous studies were addressed in the present analysis, potentially affecting the results. Finally, the lack of baseline BMD data before infection and hormonal or inflammatory biomarker measurements (e.g., IL-6, estrogen, OPG), which limits mechanistic insight. Future studies should include longitudinal follow-up to determine the causal relationship between SARS-CoV-2 reinfection and osteoporosis progression, integrate inflammatory and hormonal biomarkers to clarify mechanisms, and evaluate targeted interventions such as vitamin D supplementation and exercise rehabilitation in this population.

## 5 Conclusion

In summary, it has been found that patients with repeated SARS-CoV-2 infections had a significantly higher frequency of osteoporosis compared to the single-infected individuals. These findings offer valuable insights into the prevention of osteoporosis among older adults who have been affected by SARS-CoV-2 reinfection. Amid the ongoing COVID-19 pandemic, it is essential to closely monitor bone health in this population, particularly among older and female individuals. Maintaining adequate vitamin D levels, ensuring sufficient calcium intake, engaging in regular physical activity, and obtaining appropriate sun exposure remain key strategies for preventing osteoporosis, regardless of SARS-CoV-2 infection status.

## Data Availability

The raw data supporting the conclusions of this article will be made available by the authors, without undue reservation.
